# Birth delivery method affects expression of immune genes in lung and jejunum tissue of neonatal beef calves

**DOI:** 10.1186/s12917-017-1310-2

**Published:** 2017-12-14

**Authors:** Carla Surlis, Keelan McNamara, Eoin O’Hara, Sinead Waters, Marijke Beltman, Joseph Cassidy, David Kenny

**Affiliations:** 1 Animal and Grassland Research and Innovation Centre, Teagasc, Grange, Dunsany, Co. Meath, Ireland; 2 School of Veterinary Medicine, University College Dublin, Belfield, Dublin 4, Ireland

**Keywords:** Mode of delivery, Elective caesarean, Calf, Bovine, Lung, Jejunum, Birth

## Abstract

**Background:**

Caesarean section is a routine veterinary obstetrical procedure employed to alleviate dystocia in cattle. However, CS, particularly before the onset of labour, is known to negatively affect neonatal respiration and metabolic adaptation in humans, though there is little published information for cattle. The aim of this study was to investigate the effect of elective caesarean section (ECS) or normal trans-vaginal (TV) delivery, on lung and jejunal gene expression profiles of neonatal calves.

**Results:**

Paternal half-sib Angus calves (gestation length 278 + 1.8 d) were delivered either transvaginally (TV; *n* = 8) or by elective caesarean section (ECS; *n* = 9) and immediately euthanized. Lung and jejunum epithelial tissue was isolated and snap frozen. Total RNA was extracted using Trizol reagent and reverse transcribed to generate cDNA. For lung tissue, primers were designed to target genes involved in immunity, surfactant production, cellular detoxification, membrane transport and mucin production. Primers for jejunum tissue were chosen to target mucin production, immunoglobulin uptake, cortisol reaction and membrane trafficking. Quantitative real-time PCR reactions were performed and data were statistically analysed using mixed models ANOVA. In lung tissue the expression of five genes were affected (*p* < 0.05) by delivery method. Four of these genes were present at lower (*LAP, CYP1A1, SCN11α* and *SCN11β*) and one (*MUC5AC*) at higher abundance in ECS compared with TV calves. In jejunal tissue, expression of *TNFα, Il-1β* and *1 l-6* was higher in ECS compared with TV calves.

**Conclusions:**

This novel study shows that ECS delivery affects the expression of key genes involved in the efficiency of the pulmonary liquid to air transition at birth, and may lead to an increased inflammatory response in jejunal tissue, which could compromise colostral immunoglobulin absorption. These findings are important to our understanding of the viability and management of neonatal calves born through ECS.

## Background

Presently, caesarean section (CS) is the most common surgical procedure carried out in cattle [1, 2]. Since its introduction at the start of the twentieth century, CS is commonly employed as a final remedy to save the calf and/or the cow in complicated obstetrical cases, such as dystocia as a consequence of malpresentation or foetal oversize of the calf [2–4]. Dystocia represents the most common cause of bovine perinatal mortality, and is a significant issue for subsequent dam reproductive efficiency, with CS providing an essential role in saving both dam and calf during a difficult birth [4, 5]. The use of elective caesarean section (ECS) without an initial attempt at a natural birth, however, has become common practice in a number of breeds [5]. For example, double-muscled breeds of cattle such as the Belgian blue are typically prone to dystocia due to feto-maternal disproportion, with a high proportion of calves born through ECS. Indeed, ECS is commonly carried out in the early stages of parturition before labour has progressed [3, 5–7] In human obstetrics, ECS before the onset of labour is known to negatively affect neonatal health in early life [8–10]. Transition from intrauterine to extrauterine life is a complex process that requires a number of systemic physiological changes to take place in the neonate. Firstly, a rapid and coordinated clearance of foetal lung fluid, and the secretion of surfactants required for successful inspiration are key steps in establishing the transition from placental to gaseous exchange at birth [10, 11]. As gestational full term approaches, fluid production within the foetal lung slows, and there is also a marked increase in the pulmonary expression of sodium membrane channels, which appear to play an important role in liquid absorption [8, 10, 12]. The act of compression of the thorax during vaginal birth also appears to have a role in the expulsion of fluid during natural parturition, but to a lesser extent than the physical reabsorption of fluid at birth [9, 10]. Preterm labour and operative delivery prior to even the earliest stages of labour, have been shown to cause excessive retention of lung fluid in some mammals including preterm rabbits and foetal lambs [13].

Calves are born agammaglobulinemic, and must acquire immunoglobulin (Ig) through passive transfer from dam to neonate via colostrum consumption, which is a critical step in protection against disease [1, 14]. Passive transfer occurs in the small intestine, with reports suggesting the jejunum plays a primary role in this absorption [15–17]. Failure of passive transfer is relatively common, and is a prominent causative agent of neonatal mortality [1]. Colostrum contains additional bioactive factors, including growth factors, which the calf is able to absorb directly for the initial 20–24 h following parturition before the ability for macromolecule absorption ceases [1, 18, 19]. Previous research by Sangild 2003 has suggested that method of delivery may have an effect on the uptake of Ig in the neonatal calves and pigs, however prematurity was an additional variable alongside mode of delivery.

The aim of the research presented in this study was to establish the effect of birth delivery method on the expression of a number of key genes involved in two critical tissues- the lung and the jejunum, of neonatal Angus calves. To the author’s knowledge, this is the first study to examine transcript abundance for key genes involved in the immediate post-natal function of these two critically important tissues.

## Methods

All procedures involving animals were approved for the use of live animals in experiments by the Teagasc Animal Ethics Committee and were licensed by the Health Products Regulatory Authority in accordance with the Cruelty to Animals Act (Ireland 1897) and European Community Directive 86/609/EC.

### Animal model

Commercially purchased 18 month old oestrous synchronised Aberdeen Angus heifers (*n* = 21) were inseminated by AI with frozen thawed semen from one Aberdeen Angus sire. Foetal sex was determined in all calves at day 100 of gestation. All animals were housed during the last 2 months of gestation and allowed *ab libitum* access to a high-energy low forage diet. One week prior to predicted calving date, heifers were blocked on foetal sex to one of 2 groups; TV (calves to be delivered trans-vaginally (*n* = 10) and ECS (calves to be delivered via elective caesarean section (*n* = 11). Heifers in the TV group were induced to calve by administering 2 ml of a prostaglandin F2_α_ analogue (Estrumate, Merck), to facilitate a staggered calving schedule. Caesarean sections were carried out by an experienced veterinary surgeon using standard protocols. Local anaesthetic (Lidocaine) was used in animals undergoing caesarean section in accordance with the protocol of normal veterinary practice. All calves were euthanized within 5 min of birth by lethal injection (Dolethal (Vetoquinol), Euthatal (Merial)) administered through the jugular vein.

### Tissue sample collection

Tissue was harvested within 10 mins of slaughter using sterilized and RNase zap treated instruments. A transverse sample from the midsection of the jejunum was harvested from the small intestine from all calves, washed in DPBS and snap frozen in liquid nitrogen. Lung tissue was sampled from the centre of the right lung from all animals in a consistent manner. Tissue samples were washed in DPBS, and subsequently snap frozen in liquid nitrogen until analysis.

### Histology

In order to ensure consistency in cellular content for all samples, histological staining and imaging was conducted prior to transcriptomic analysis. Tissue was sampled from an area approx. 2 cm from the center of the right lung in each animal and placed immediately in 10% buffered formalin (pH 7.4). After 24 h, the samples were processed using an automated processor (Tissue-TEK VIP, Sakura Finetek) and embedded in paraffin wax. Sections of 5 μm were cut using a microtome (Leitz 1512), and mounted onto glass slides. Samples were stained using a haematoxylin and eosin stain and were visualized using Image-Pro Plus software (Version 5, Media Cybernetics) [20].

### RNA extraction

Total RNA was isolated from jejunum and lung tissue samples (TV *n* = 8, ECS *n* = 9) using the Qiagen RNeasy plus universal mini kit (Qiagen, UK), according to manufacturer’s instructions. We failed to recover samples, due to logistical reasons, from three calves delivered by TV and one calf delivered by ECS. Approximately 75 mg of frozen tissue was used for RNA extraction. RNA quality was determined by measuring the absorbance at 260 nm using a Nanodrop spectrophotometer ND-100, to ensure all samples had an absorbance of between 1.8 and 2.0 (Nanodrop Technologies, Wilmington, DE, USA). RNA quality was assessed on the Agilent Bioanalyser 2100 (Agilent Technologies Ireland Ltd., Dublin, Ireland) using the RNA 6000 Nano lab chip kit, with RIN value of between 8 and 10 were deemed to be of sufficiently high quality. RIN values of samples that did not meet these requirements were further purified using the RNA clean and concentrate kit (Zymo Research, UK), according to manufacturer’s instructions.

### Complementary DNA synthesis

cDNA was synthesized using a High Capacity cDNA Reverse Transcription kit (Applied Biosystems, Foster City, CA, USA) according to the manufacturer’s instructions. 2 μg of total RNA from each sample was reverse transcribed into cDNA using MultiScribe reverse transcriptase. The converted cDNA was quantified by absorbance at 260 nm and stored at −20 °C for subsequent analyses.

### Primers

Specific genes involved in immune response and pulmonary liquid-gas transition in neonates were targeted in this study and are outlined in Table 2. All primers for real-time PCR were designed using Primer3web (http://primer3.ut.ee/). Primers were then subjected to BLAST analysis (http://www.ncbi.nlm.nih.gov/) to confirm their specificity and ensure that they were homologous to the bovine sequence. Details of primer sets used in this study are listed in Tables 1 and 2. Primers for reference and target genes were commercially synthesized (Sigma-Aldrich Ireland, Dublin, Ireland). A total of 13 genes were chosen for target in the lung tissue, with the majority of the genes selected to examine alterations in the ability of ECS delivered calves to produce the necessary surfactants and mucin proteins for normal lung function. A number of immune and stress related genes such as *LAP* and *CYP1A1* were also chosen for target, to investigate a potential effect of both by ECS on the stress response and subsequent immune response in neonatal calves. In addition to these, two sodium channel genes were also chosen, to examine whether or not there could be a potential molecular basis for fluid retention in the ECS delivered neonate. A total of 15 genes were chosen for target in jejunal tissue including a number of interleukins to examine alterations to the immune response, and a number of receptors including a component of the FC receptor complex, which plays an important role in Ig absorption from colostrum. A number of genes involved in extracellular matrix structure were also examined, to identify any structural conformation changes in the jejunum of ECS delivered calves that may affect Ig absorption. Five housekeeping genes were selected from commonly used reference genes for lung and jejunum tissue: β-actin (*ACTB*), glyceraldehyde-3-phosphate-dehydrogenase (*GAPD*), 40s Ribosomal Protein S9 (RSP9), Ubiquitously Expressed (FAU) and Hypoxanthine Phosphoribosyltransferase 1 (HPRT1). The gene expression levels were measured by real-time qPCR, and the expression stabilities were evaluated by the M value of geNorm.

**Table 1:** Sequences of oligonucleotide primers used for qPCR analysis of lung tissue

Gene ID^a^	Primers (5′-3′)	Accession No.	Amplicon length
*MUC5AC*	Forward: CAGTACAGAGTGCATGGGGA Reverse: TTCACAAACACCTCCCCACT	XM_015470102.1	185
*MUC5B*	Forward: AAAACGCCCTTCACCTTCAC Reverse: TGCCTCAGGTTCTCGAATGT	XM_015470101.1	176
*LAP*	Forward: CCCTGGAAGCATGAGACAGA Reverse: TTTCTGACTCCGCATCCAGT	S76279.1	109
*SP-A*	Forward: TGGGGAGGCATCTTGTTAGG Reverse: TGTTCATCAGCAGGCAGGTA	NM_001077838.2	194
*SP-B*	Forward: GACATGTGGAAGCCGATGAC Reverse: TGAGTCCTGGAAAATGGCCT	NM_001075311.2	92
*SP-C*	Forward: GAGATCCAGGAGCAAAGGGT Reverse: CCTCCCACAGTCCCATTTCT	NM_174462.4	95
*SP-D*	Forward: CCTGTACCCTGGTCATGTGT Reverse: AGCAGAGCCATTGTCTCCTT	NM_181026.2	179
*CYP1A1*	Forward: GTCACAACTGCCCTTTCCTG Reverse: AAAGGAGGAGTGTCGGAAGG	XM_005192890.3	180
*CYP1A2*	Forward: TCCTCTTCCTGGCCATCTTG Reverse: CAGAACGCCAGCAACTTCTT	XM_015468527.1	165
*ABCA3*	Forward: GAGCACACCTTCAACCACAG Reverse: AAAGGAGCCTGTCTGAGTCC	NM_001113746.1	108
*SCN11A*	Forward: ATGCTGGCTTTAATCTGCGG Reverse: CCTGGAAGCACGAATGGATG	NM_174598.3	177
*SCN11B*	Forward: CTGAAGGACCTGGACGAACT Reverse: TTGATAAAGACCAGGGGCGT	NM_001098075.1	128
*SGK-1*	Forward: GGCTCGATTCTATGCTGCTG Reverse: ACGTTGTGCCATTGTGTTCA	NM_001102033.1	121

^a^ *MUC5AC* mucin 5 ac, *MUC5B* mucin 5B, *LAP* lingual antimicrobial peptide, *SP-A* Surfactant protein A, *SP-B* Surfactant protein B, *SP-C* surfactant protein C, *SP-D* surfactant protein D, *CYP1A1* cytochrome P45 family 1 subfamily A member 1, *CYP1A2* cytochrome P450 family 1 subfamily A member 2, *ABCA3* ATP binding cassette subfamily A member 3, *SCN11A* Sodium channel voltage-gated type 2 alpha subunit, *SCN11B* Sodium channel voltage-gated type 2 beta subunit, *SGK-1* Serum/glucocorticoid regulated kinase 1

**Table 2:** Sequences of oligonucleotide primers used for qPCR analysis of jejunum tissue

Gene ID^a^	Primers (5′-3′)	Accession No.	bp^2^
*NR3C1*	Forward: TACAGGCAGCAATGGTCTCA Reverse: AAGAGGGTGGTCATTCTGGG	NM_001206634.1	154
*MUC1*	Forward: CCCAACTCTGTTCTGGGCTA Reverse: TTCCAGCCAGTATTCCAGCA	AJ400824.1	163
*MUC2*	Forward: TGCTACTACGTGCTGACCAA Reverse: ACGTTCTTCTTGTTGTCGGC	XM_010806231.2	131
*TNF- α*	Forward: CGTGGACTTCAACTCTCCCT Reverse: GGACACCTTGACCTCCTGAA	NM_173966.3	179
*Il-1 β*	Forward: CCAGCTGCAGATTTCTCACC Reverse: TCACACAGAAACTCGTCGGA	NM_174093.1	195
*il-4*	Forward: TGCCCCAAAGAACACAACTG Reverse: GAACAGGTCTTGCTTGCCAA	NM_173921.2	144
*Il-6*	Forward: ACTTCTGCTTTCCCTACCCC Reverse: TGTCGACCATGCGCTTAATG	NM_173923.2	121
*VIP*	Forward: TCGACTCCCAGGACTTCAAC Reverse: GAGAAGAGCACGCTGAACAG	NM_173970.3	148
*FCGRT*	Forward: ACTATCGCTCGCTCCAGTAC Reverse: CCTGCGCCCGTAGATTATTG	NM_176657.1	126
*B2M*	Forward: GTTCACTCCCAACAGCAAGG Reverse: TCTCGATGGTGCTGCTTACA	NM_173893.3	109
*RAB11A*	Forward: GCAACAAGAAGCATCCAGGT Reverse: TAAGGCACCTACAGCTCCAC	NM_001038162.2	120
*RAB25*	Forward: AGCTGAGAGTTGAGGGCATT Reverse: TCGGCTCTGTTTCCCATCTT	NM_001017936.1	102
*STX3*	Forward: GAGCAGCATCAAGGAGCTTC Reverse: TACCAATTTCTTCCGGGCCT	NM_001101971.1	181
*pIg*	Forward: GGTGTGCTGGTTCCTTCTTG Reverse: ATAAGCTGCAGGTGGGAACT	NM_174143.1	93
*MYO5B*	Forward: GAAGCAATACCGCATGCAGA Reverse: TTCTGGATGATGGTGGCCTT	XM_015469176.1	147

^a^ *NR3C1* Nuclear receptor subfamily 3 group C member 1, *MUC1* Mucin 1, *MUC2* Mucin 2, *TNFα* Tumor necrosis factor alpha, *IL-1β* Interleukin 1 beta, *IL-4* Interleukin 4, *IL-6* Interleukin 6, *VIP* Vasoactive intestinal polypeptide, *FCGRT* fc fragment of Ig receptor and transporter, *B2M* Beta −2- microglobulin, *RAB11A* Ras related protein Rab11a, *RAB25* Ras related protein RAB25, *STX3* Syntaxin 3, *pIg* Polymeric immunoglobulin receptor, *MYO5B* Myosin VB

### Real-time quantitative PCR

Real-time quantitative PCR was carried out using the ABI 7500 Fast real-Time PCR System with SYBR green Master Mix (Applied Biosystems, Warrington, UK). Reactions were carried out in a 96-well plate (Applied Biosystems, Warrington, UK) and prepared in a total volume of 20 μl, with 2 μl cDNA, 10 μl SYBR green master mix, 7 μl of nuclease free H20 and 1 μl of 5 μM forward and reverse primer mix. Optimal cDNA concentration, primer efficiencies and concentrations were determined. Thermal cycling conditions applied to each assay consisted of an initial denaturation step at 95 °C for 15 min followed by 40 cycles of denaturation at 95 °C for 5 s and annealing and extension at 60 °C for 40 s. At the end of each cycle, SYBR green fluorescence was detected to monitor the quantity of PCR product. The efficiency of the qPCR reaction was calculated for each gene by creating a standard curve from two-fold serial dilutions of cDNA. Only primers with PCR efficiencies between 90 and 110% were used. The software package GenEx 5.2.1.3 (MultiD Analyses AB, Gothenburg, Sweden) was used for efficiency correction of the raw Ct values, normalisation to the reference genes, and calculation of quantities relative to the average Ct value for each gene.

### Statistical analyses

Data were checked for normality using the UNIVARIATE procedure of Statistical Analysis Software (SAS, version 9.3). Data were transformed by raising co-efficients to the appropriate power of λ using the TransReg procedure when appropriate, and subsequently analysed using mixed model methodology within the MIXED procedure of SAS. The Tukey critical difference test was performed to determine the presence of statistical differences, with *P* < 0.05 was considered significant.

## Results

### Histology of lung tissue

**Fig. 1:**
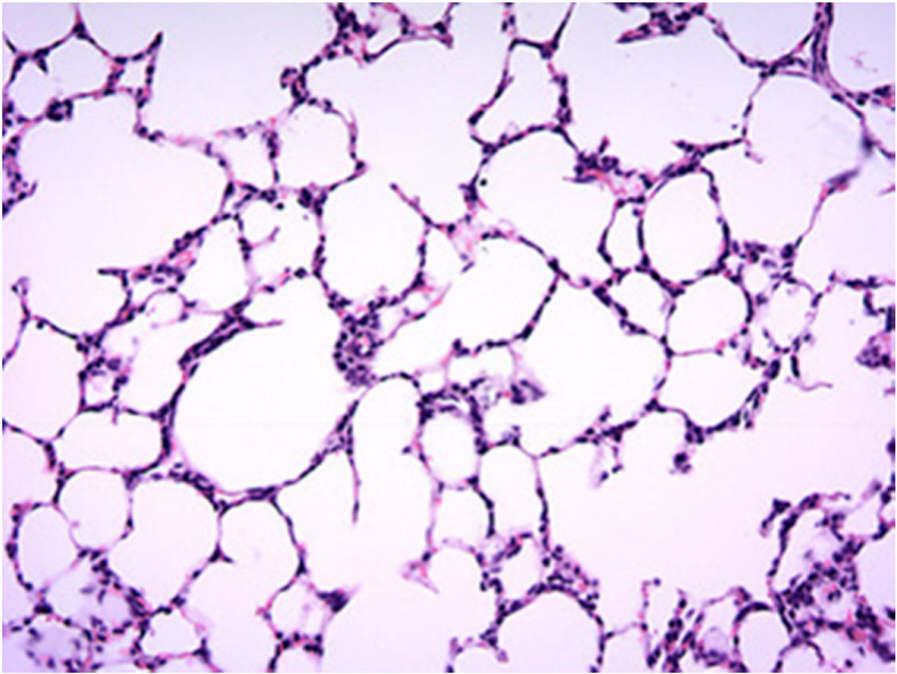
Histology findings for lung tissue taken from the centre right lobe of the right lung in calves delivered natural by vaginal birth at ×20 magnification

**Fig. 2:**
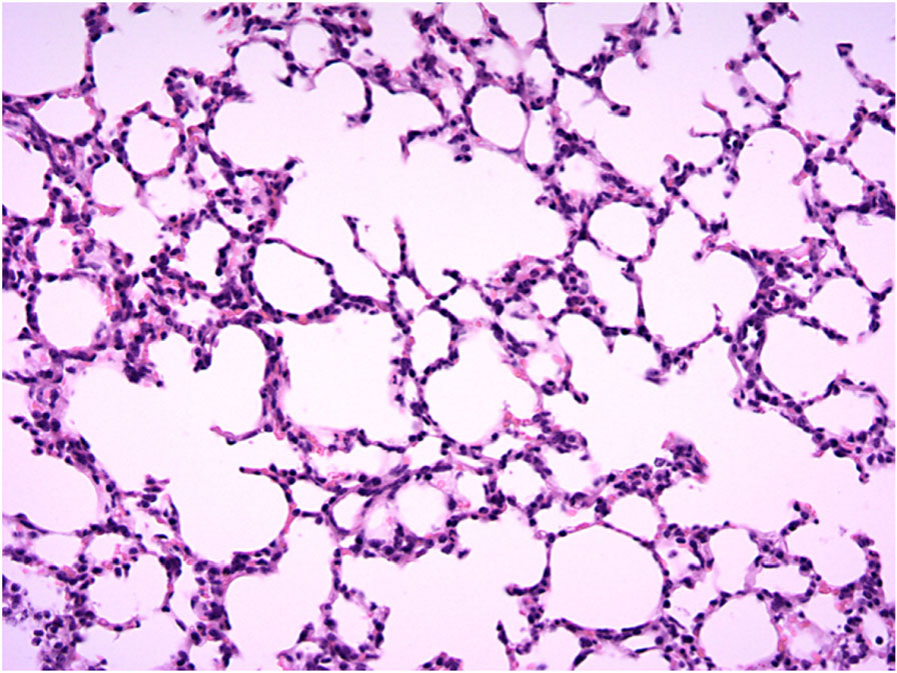
Histology findings for lung tissue taken from the centre right lobe of the right lung in calves delivered by elective caesarean section at ×20 magnification

Tissue was analysed to ensure samples taken were of sufficient similarity prior to transcriptomic analysis. A representative image from the lung tissue sampled from trans-vaginally delivered and elective caesarean delivered calves can be seen in Figs. 1 and 2 respectively. Following staining and visualisation, it was determined that samples were of a consistent cellular similarity for transcriptional analysis.

### Differential expression of genes in lung and jejunum tissue

#### Lung tissue

A total of 13 genes were chosen for analysis in the lung tissue of the TV and ECS delivered calves (See Table 1 for primer list) and the effect of delivery method on transcript abundance is outlined in Table 3. There was a statistically significant effect of delivery method on the relative transcriptional abundance of 5 genes from the 13 chosen. Transcript abundance for *LAP,* an antimicrobial peptide, was 2.06 fold lower in ECS compared with TV calves (*p* = 00.75; Table 4). Similarly *CYP1A1,* a member of the detoxifying cytochrome P450 superfamily, was 3.95 fold lower in ECS calves (*p* = 0.0016). *MUC5AC*, which encodes a secreted mucin protein, was present at higher levels of abundance (2.55 fold difference; *p* = 0.0021; Table 4) in the ECS delivered calves. Two genes which encode for cellular sodium voltage gated channel components were lower in abundance in calves delivered via ECS (*SCN11a*; 2.73 fold; *p* = 0.0002 and *SCN11b*: 1.72 fold; *p* = 0.0051).

**Table 3:** Effect delivery method on the expression of genes in calf lung and jejunum tissue. Table highlights expression levels of genes in ECS delivered calves relative to the TV delivered controls

Symbol	Gene annotation	Fold change^a^	*P* value^2^
Lung Tissue			
*LAP*	Lingual antimicrobial peptide	−2.06	**0.0075**
*SP-A*	Surfactant protein A	−1.33	0.2890
*SP-B*	Surfactant protein B	−1.20	0.5915
*SP-C*	Surfactant protein C	−1.29	0.2616
*SP-D*	Surfactant protein D	1.47	0.2065
*SCN11A*	Sodium voltage-gated channel alpha subunit 11 alpha	−2.73	**0.0002**
*SCN11B*	Sodium voltage-gated channel beta subunit 11 beta	−1.72	**0.0051**
*SGK1*	Serine/threonine-protein kinase 1	1.29	0.8690
*MUC5AC*	Mucin 5 subtype AC	2.55	**0.0021**
*MUC5B*	Mucin 5B	−2.07	0.6330
*ABCA3*	Bovine ATP-binding cassette sub-family A member 3	−1.31	0.4254
*CYP1A1*	Cytochrome P450 family 1 subfamily A member 1	−3.95	**0.0016**
*CYP1A2*	Cytochrome p450 family 1 subfamily 1 member 2	−1.42	0.2811
Jejunum Tissue			
*IL-6*	Interleukin 6	1.23	**0.0018**
*IL-4*	Interleukin 4	1.77	0.162
*IL1-β*	Interleukin 1 beta	1.92	**0.0013**
*FCGRT*	FC fragment of Ig receptor and transporter	1.24	0.1643
*MYO5B*	Myosin 5B	−1.41	0.2863
*VIP*	Vasoactive intestinal polypeptide	1.08	0.6519
*MUC1*	Mucin 1	−1.76	0.6519
*MUC2*	Mucin 2	−1.80	0.1456
*TNFα*	Tumour necrosis factor alpha	1.92	**0.001**
*B2M*	Beta-2-microglobulin	1.28	0.3555
*NR3C1*	Nuclear receptor subfamily 3 group C member 1	−1.05	0.1001
*Rab11a*	Ras related protein Rab11a	1.38	0.4954
*Rab25*	Ras related protein Rab25	1.02	0.3995
*STX3*	Syntaxin 3	−1.08	0.3794
*pIg*	Polymeric Immunoglobulin receptor	1.13	0.6741

P values in bold indicate significant change to the transcriptional levels of the gene

^2^ *P* values were corrected (Bonferroni correction)

^a^Fold change represents observed reduction or increase in abundance of gene in CS delivered calves

**Table 4:** Sequences of oligonucleotide primers selected as reference genes for qPCR analysis

Gene ID^a^	Primers (5′-3′)	Accession No.	Amplicon length
*Act- β*	Forward: CGGCATCGAGGACAGGAT Reverse: CATCGTACTCCTGCTTGCTGAT	NM_173979.3	169
*GAPDH*	Forward: CCTGCCCGTTCGACAGATA Reverse: GGCGACGATGTCCACTTTG	NM_001034034.1	150
*FAU*	Forward: CCGCATGCTTGGAGGTAAAG Reverse: CACAACATTGACAAAGCGCC	NM_174731.3	154
*RPS9*	Forward: CCGCATGCTTGGAGGTAAAG Reverse: CACAACATTGACAAAGCGCC	NM_001101152.2	188
*HPRT1*	Forward:GGATTACATCAAAGCACTGAACA Reverse: CATTGTCTTCCCAGTGTCAATT	NM_001034035	194

^a^ *Act- β* β-actin, *GAPDH* Glyceraldehyde-3-Phosphate Dehydrogenase, *FAU* Finkel-Biskis-Reilly Murine Sarcoma Virus (FBR-MuSV) Ubiquitously Expressed, *RPS9* Ribosomal Protein S9, *HPRT1* Hypoxanthine Phosphoribosyltransferase 1

#### Jejunum tissue

Fifteen genes were chosen for comparative quantitative analysis of jejunal function between calves delivered by caesarean section compared with natural transvaginal birth. Differences in transcript abundance between the treatments for three out of the 15 genes analysed reached statistical significance Table 4. Two of the genes were members of the Interleukin superfamily; *Il-6* (1.23 fold increase; *p* = 0.0018; Table 4) and *IL-1β* (1.77 fold increase; *p* = 0.0013, Table 4) were higher in calves delivered by ECS compared with TV calves. An additional gene from the interleukin superfamily, *IL-4* was present at an increased transcript abundance in CS calves, with a tendency towards significance (1.13 fold increase; *p* = 0.057; Table 4). There was a tendency towards significance (*p* = 0.10) on the effect of birthing method on *NR3C1*, present at lower abundance in the calves delivered by ECS. This gene encodes for a receptor for glucocorticoid, a corticosteroid hormone which is heavily involved in neonatal adaptation during birth and early life.

## Discussion

### Lung function in calves delivered by elective caesarean section

#### Fluid absorption

The transition of the lungs from an aqueous environment to one of oxygen is arguably the most vulnerable and critical adaptation phase in neonatal development immediately following birth. The neonate must rapidly assume responsibility for oxygenating its own blood from atmospheric air, by clearance of fluid from the lung cavity and by the production of sufficient amounts of glycerophospholipid-rich surfactant lipoproteins [21, 22]. The lung, rather than the amniotic sac itself, is the source of liquid in the foetal lung [10, 23]. As parturition approaches, the rate of liquid formation and the volume of liquid within the lung decreases [10]. The first challenge for the neonate to overcome upon delivery is to rapidly clear air spaces of the remaining fluid [24]. The passage of the neonate through the birth canal is thought to be important for aiding the expulsion of liquid from the lungs, due to the “Vaginal squeeze”, and calves born by caesarean before the onset of labour are completely deprived of this [10]. Hormonal changes immediately prior to labour, which continue throughout are thought to play an important role in preparing the lungs for liquid removal and for aerobic respiration, in particular, the release of epinephrine from the adrenal medulla. Amiloride sensitive sodium transport is a key event in the trans-epithelial movement of alveolar fluid in the lungs, which is enabled by sodium channels lining the epithelial layer [10, 25]. At late stage gestation, an increased expression of epithelial sodium channels has been demonstrated in mammals, with many authors reporting much lower expression of sodium channel subunits in preterm human neonates suffering from respiratory distress [10, 26]. These sodium channel subunits were also shown to be present at significantly reduced levels of abundance in neonates that had been delivered via elective caesarean section [26]. This reduction is most likely due to the lack of the surge in hormones normally induced by labour. Cortisol, one of the main labour induced hormones is known to activate these pumps following parturition [11] The clearance of fluid by the action of these sodium channels involves a two-step process: firstly, the passive movement of sodium (Na^+^) from the lumen, across the apical membrane and into the cell through the sodium voltage gated channels, followed by the active extrusion of Na^+^ from the cell, across the basolateral membrane and into the serosal space [25]. The remaining fluid is then rapidly reabsorbed as water and the lungs cleared [27] The sodium channel ENaC is comprised of three subunits, and we demonstrated a significant decrease in the abundance of the alpha and beta subunits in the calves delivered by elective caesarean section. The decreased relative abundance of these subunits indicates a reduced availability of sodium channels in the lung epithelial layer of calves delivered by ECS, and such channels may significantly affect transition of the calf at birth. The reduction in the transcriptional abundance of these genes may be due to the lack of the hormonal surge in the prepartum period, and this, combined with the lack of the mechanical fluid clearance mechanisms during the uterine contractions of a vaginal delivery, may lead to a much slower clearance of fluid from the lungs of ECS delivered calves and could affect potentially affect future pulmonary function [10, 12, 28].

#### Secretion of normal lung proteins

As the end of gestation approaches, foetal lung fluid declines in preparation for delivery. At this time, the production of surfactant proteins initiates, with labour prompting the secretion of these proteins into the remaining foetal lung fluid, which increases the overall surfactant concentration of the lung [29]. Without the onset of labour, the initiation of this surfactant secretion does not occur, meaning that the overall concentration does not reach the necessary levels needed for rapid transition at birth [11]. ECS deliveries before the prepartum increase of hormones also results in a reduction in the expression of additional proteins such as antioxidants and does not occur at its peak and necessary level [30]. This was highlighted in our study, as we showed an almost four fold reduction to the relative abundance of *CYP1A1*. The lungs are a major target for all inhaled toxins, and also of endogenously derived cellular toxins such as ROS. This gene is part of the cytochrome p450 super family of isoenzymes, which are capable of detoxifying both endogenous and exogenous toxins and has been shown to play a critical role in protection against hyperoxic lung injury by reducing lipid peroxidation and oxidative stress in both human and murine studies [31, 32]. Hyperoxia is commonly seen in both preterm infants and those delivered by ECS suffering from respiratory difficulty [31]. The reduced abundance in transcript levels of this gene may indicate that calves delivered by ECS may be more susceptible to toxins and hyperoxic stress, which could affect transition at birth and in the subsequent days that follow. The expression levels of two Mucin genes were also examined for alterations in their abundance in the ECS delivered calves.

#### Lung immunity

Transcript abundance for *LAP,* an antimicrobial peptide (AMP) found in the lung, was more than two-fold lower in the calves delivered by ECS compared with TV. As a member of the β-defensin group of AMPs, LAP plays a critical role in protection against opportunistic invading pathogens. Defensins have been shown to exhibit a developmentally regulated expression from foetus to neonate, with much lower expression found in foetal sheep and humans in comparison to levels expressed in their neonatal counterparts [33, 34]. Natural trans-vaginal delivery is associated with a steady increase in stress in the neonate, important for the expression of genes critical to adaptation. For example, an increased expression of proinflammatory cytokines during labour of both term and preterm neonates causes a correlated increase in the levels of SP-A produced [22]. Here, we suggest that the significantly lower levels of *LAP* could be due to the absence of a rise in the neonatal stress levels associated with natural TV delivery. The lower level of transcriptional abundance demonstrated here could have serious implications leading to an inactivated and impaired lung immunity in the ECS delivered calves [35]. An additional gene, *MUC5AC,* was found to be significantly higher in the calves delivered by ECS. *MUC5AC* encodes the protein backbone of the MUC5AC glycoprotein. Its presence here at higher levels of expression in the calves delivered by section indicates a possible excess production of mucous, which is symptomatic of many lung disease states, including bronchial hyperactivity in asthma [36] Mucin proteins also serve as binding points for bacteria, potentially problematic for the already impaired immunity of ECS delivered calves [27, 37].

### Jejunal gene expression and implications for immunoglobulin absorption

Immediately following birth, the new-born must respond to a huge influx of potentially dangerous pathogens [1, 38]. While passive immunity exists in humans at birth, calves, like other ruminant neonates and some other species like pigs, are born agammaglobulinemic [18]. Transfer of Ig to the foetus in cows is not possible due to epitheliochorial placentation which interposes large numbers of epithelial layers between the maternal and foetal blood supplies [39, 40]. Immune transfer of immunoglobulins (Ig) and other bioactive factors such as lysozyme and growth hormones must occur rapidly after birth through passive transfer from colostrum to the neonate [1, 41]. Failure of sufficient passive transfer is thought to be a major contributing factor in perinatal mortality in calves, and the aim of the expression analysis in this study was to ascertain whether there was evidence that delivery by ECS prior to the normal foetal cortisol surge may affect the immune response of the jejunum or on the potential uptake of Ig within the intestinal tract [18].

To examine a possible effect of mode of delivery on the absorption of Ig through passive transfer in the neonatal calves, a number of genes with proposed function in gastrointestinal absorption were selected. *FCGRT,* encodes for the alpha chain of the bovine receptor FcRn. This receptor plays a critical role in the absorption of Ig through passive transfer in calves [42] There was no observed difference in the expression of this gene between the ECS and TV delivered calves. The expression of polymeric immunoglobulin receptor (pIg) was also analysed, with no significant difference to its relative abundance observed between the ECS and TV delivered calves. pIg is a receptor present in epithelial cells along the intestinal tract which is known to play a vital role in Ig passage through the epithelial layer [43, 44]. The absence of a significant change to the abundance of these Ig receptors suggests that mode of delivery does not affect the absorption of Ig, at least at this early time point. Upon colostrum intake, a number of genes involved in cell growth and the immune response are known to become highly expressed, and possibly colostrum ingestion also influences the expression of Ig absorption associated genes, which may have been apparent had the calves been permitted to ingest colostrum [45, 46]. A number of genes involved in intracellular trafficking with possible roles in the transport of Ig following uptake by pinocytosis were also examined for alterations to their relative transcriptional abundance [14, 18]. *Rab11a* and *Rab25,* which both encode Ras-related proteins involved in the absorption of molecules such as calcium through endocytosis in the intestinal epithelial membrane were also examined for changes in their expression levels [47, 48]. Neither gene was changed at a level that was deemed statistically significant, but possibly would have presented at altered levels in calves following colostrum absorption.

#### Immune gene expression in the neonatal jejunum

Three genes, out of the 15 in total that were analysed for quantitative variations between the calves delivered either naturally by TV or by ECS, were present at higher abundance in the latter; *Il-6, Il-1β* and *TNFα.* All three genes are immune related genes, and members of the proinflammatory cytokine group. Proinflammatory cytokines are known to play an important role during labour and delivery, such as ripening of the cervix, and are critical to immune protection of the neonate in response to influx of gastrointestinal pathogens [49, 50]. The birth process itself has recently received attention as a possible stimulus for both immediate and long term disease susceptibility in both humans and in animals, with the altered immune response in jejunal tissue observed in our study highlighting this as a possibility [51]. There have been conflicting results with regard to the effect of mode of delivery on the abundance of cytokines, with similar expressional analysis demonstrating opposite results in both cord and blood samples. For example, Ly et al. (2006) demonstrated increased abundance of *Il-1β*, *Il-6* and *IFNγ* in cord blood samples of human neonates that had been delivered before labour by ECS. In contrast, a similar study using cord blood samples of neonatal humans born by ECS found these genes plus *Il-4* and *TNFα,* were found at a lower level of abundance [52]. An additional study found further conflicting results, with quantitative analysis of plasma samples showing an increase in the abundance of *Il-6* but a reduction to the abundance of *Il- 2* in neonates delivered by ECS [53]. The increased abundance of certain proinflammatory cytokines including the three that were increased in the calves delivered before the onset of labour in our study, have been linked with certain allergenic diseases in later life, including asthma [52]. Cytokines are also known to play an interactive role between the immune and the neuroendocrine system, with the expression of certain types (e.g. *Il-1β* and *TNFα*) known to activate the hypothalamic-pituitary axis, also acting on the central nervous system [53]. During normal delivery, the neonate is subjected to increased levels of stress over time, compared to the stress caused by ECS, which is more immediate and short-term, and this may be the cause of the heightened expression of these pro-inflammatory cytokines [35]. A previous study observing expression of cytokines in human neonates suggested that altered abundance in the transcript levels of these cytokines could be due to the use of operative anaesthetic, both regional and general, during the procedure, which influences expression within the foetus before birth [54]. However, conflicting results were demonstrated in an additional comparative mode of delivery study using neonatal piglets, where an increased abundance of *TNFα* was seen in piglets delivered by caesarean section, but delivery was carried out immediately after stunning of the dams in the trial, with no anaesthesia or analgesia used [55]. The increase in abundance of these genes could potentially be due to an overall stress response in the calves when delivered so abruptly before endogenous hormonal preparation, but is unlikely as the process of natural labour and birth is thought to cause the greatest stress to both neonate and dam [53]. The functional integrity of the intestine is critical to the immunocompetence of the bovine neonate. Immediately after birth, the neonatal animal must, alongside other adaptations rapidly required for successful transition, adjust from an aqueous environment sterile environment to that of an atmospheric one laden with opportunistic pathogens. The lack of passage through the birth canal poses an immediate threat to neonates born by caesarean section, particularly exaggerated in those born before the pre-labour hormonal surge where a compromised immune response as observed here may mitigate against the animals’ immunocompetence [56].

Two genes from the mucin family, *MUC1* and *MUC2* were also examined for changes to their relative abundance in the TV compared with ECS calves. A healthy epithelial mucin layer in the gastrointestinal tract is vital for many functions including lubrication of food, maintenance of a physical barrier protection against pathogens, and for provision of a permeable gel layer through which gaseous and nutrient exchange can take place [57]. Despite their putative roles in the absorption of bioactive factors in colostrum, we failed to observe an effect of mode of delivery on the relative expression of these genes [57–59].

## Conclusion

In the current study, we provide some evidence that lung tissue in calves delivered by ECS may be compromised for efficient transition to neonatal life. Specifically, we observed reduced abundance of two sodium channel subunits, critical for rapid clearance of fluid from the lungs. Comparison of gene expression for the jejunal tissue between the two birthing processes employed highlighted a number of alterations to the ECS delivered calves that could have serious consequences for subsequent post-natal health. While we did not observe direct evidence that the potential for immunoglobulin absorption may be negatively affected by ECS in calves, it is possible that such effects may be manifested at a later stage following colostrum ingestion.

The work here highlights the potential risks to neonatal calf health following elective caesarean section before the onset of labour. Further work is required to ascertain whether or not colostrum immunoglobulin uptake is compromised, and to examine whether latent effects of birth process on the functionality of the immune system exist. These data also offer value to understanding potential negative effects from the use of elective caesarean section procedures in humans.

## Abbreviations

AI: Artificial insemination
Ct value: Cycle threshold value
DPBS: Dulbecco’s phosphate-buffered saline
ECS: Elective caesarean section
Mins: Minutes
PCR: Polymerase chain reaction
qPCR: Quantitative polymerase chain reaction
RIN: RNA integrity value
TV: Transvaginal delivery
